# Unraveling the autoimmune architecture of pemphigus: from B-cell depletion to network-based immune engineering

**DOI:** 10.3389/fimmu.2026.1844632

**Published:** 2026-07-07

**Authors:** Pedram Shafiei-Jahani, Xin Li, Amitis Akbari, Anissa Vera, Emily Haniff, Ryan Carlisle, Omid Akbari, Vanessa Holland

**Affiliations:** 1 David Geffen School of Medicine at University of California, Los Angeles (UCLA), Los Angeles, Los Angeles, CA, United States; 2 Department of Molecular Microbiology and Immunology, Keck School of Medicine of University of Southern California (USC), Los Angeles, CA, United States; 3 Division of Dermatology, Department of Medicine, University of California, Los Angeles (UCLA), Los Angeles, Los Angeles, CA, United States

**Keywords:** autoimmune blistering disease, IgA pemphigus, immune tolerance, paraneoplastic autoimmune multiorgan syndrome, paraneoplastic pemphigus, pemphigus, pemphigus foliaceus, pemphigus vulgaris

## Abstract

Pemphigus encompasses autoimmune blistering diseases driven by autoantibodies to desmosomal proteins. Disruption of keratinocyte adhesion produces acantholysis and epidermal fragility. Anti-CD20 B-cell depletion and IgG lowering strategies have transformed care, but many patients relapse, some variants respond incompletely, and durable drug-independent tolerance remains uncommon across variants. These gaps highlight that pemphigus is not a simple linear B-cell disorder, but an autoantibody disease sustained by a network linking the innate immunity, the adaptive lymphocytes, and the stromal niches. In this review, we focus on pemphigus vulgaris, pemphigus foliaceus, paraneoplastic pemphigus/paraneoplastic autoimmune multiorgan syndrome, and IgA pemphigus as representative variants within this network. We place these entities within a conceptual type 1, type 2, and type 3 immunity framework and summarize how central and peripheral B-cell tolerance can fail. We further consider current and emerging therapies as partial interventions within this network and outline next-generation strategies that modulate innate circuits, rewire survival pathways, restore antigen-specific regulation, and shift the adaptive autoimmune response toward a more tolerogenic state. Framing pemphigus in this way may help move the field from empiric immunosuppression toward precise immune engineering with the goal of durable immune reset and long-term drug-independent remission.

## Introduction

**Figure 1:**
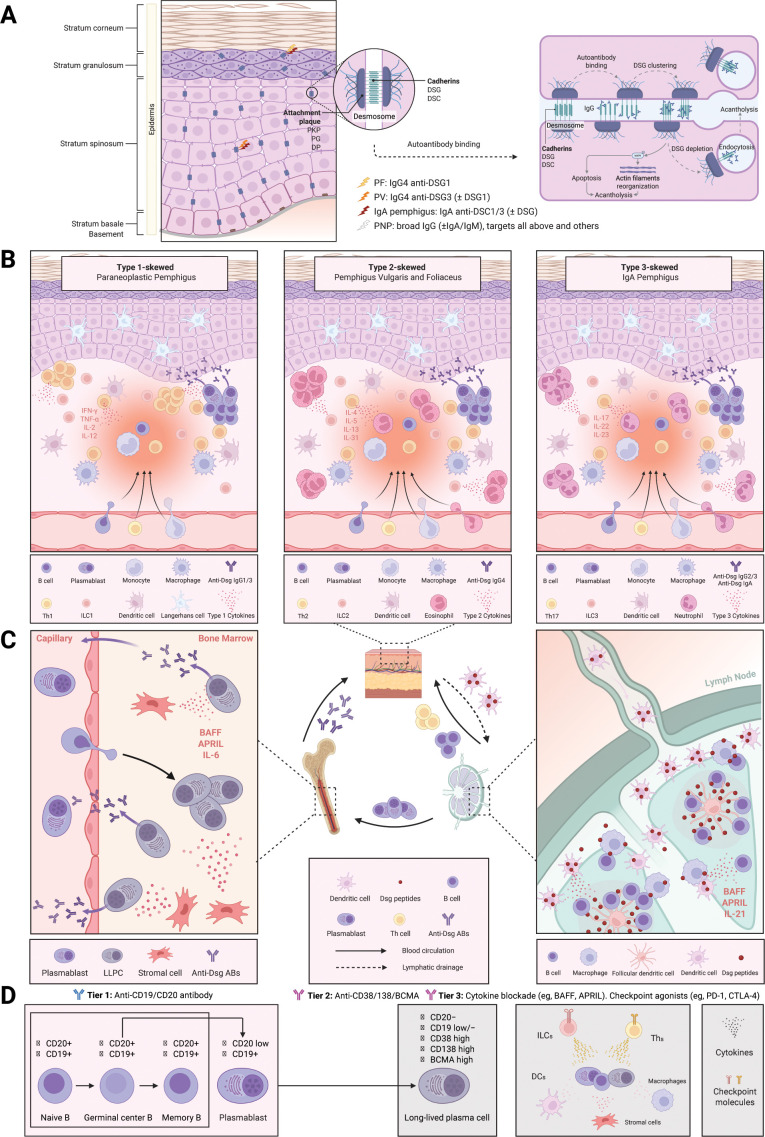
Autoimmune architecture of the pemphigus spectrum and network-based therapeutic nodes. **(A)** Desmosomes maintain keratinocyte adhesion through desmoglein (DSG) and desmocollin (DSC) cadherins linked to plakoglobin (PG), plakophilins (PKP) and desmoplakin (DP). Pemphigus autoantibodies engage DSG, drive DSG clustering and endocytosis, activate mitogen-activated protein kinase (MAPK) signaling with actin remodeling, reduce surface DSG and culminate in acantholysis. Representative autoantibody profiles across pemphigus foliaceus (PF), pemphigus vulgaris (PV), IgA pemphigus and paraneoplastic pemphigus/paraneoplastic autoimmune multiorgan syndrome (PNP/PAMS) are shown. **(B)** A conceptual type 1, type 2 and type 3 immunity framework positions dominant immune tendencies across pemphigus variants within distinct cytokine milieus and effector programs. PNP/PAMS is enriched for type 1 features with IFN-γ, TNF-α, IL-2 and IL-12 and predominance of T helper 1 cells and innate lymphoid cells 1. PV and PF are depicted as relatively type 2-skewed based on emerging immune-signature data, with IL-4, IL-5, IL-13 and IL-31 and relative involvement of T helper 2 cells, innate lymphoid cells 2 and eosinophils. IgA pemphigus is enriched for type 3 features with IL-17, IL-22 and IL-23 and predominance of T helper 17 cells, innate lymphoid cells 3 and neutrophils. **(C)** A distributed autoreactive network connects skin, lymph node, circulation and bone marrow. Antigen sampling and presentation support germinal center reactions, plasmablast output and anti-DSG antibody production, whereas long-lived plasma cells persist within stromal niches supported by BAFF, APRIL and IL-6 in bone marrow and by BAFF, APRIL and IL-21 in lymph node, sustaining autoantibody production and supporting relapse after B cell depletion. **(D)** Tier 1 targets the CD19+ and CD20+ upstream B-cell compartment and reflects current clinical practice. Tier 2 represents emerging strategies that extend depletion to plasmablasts and long-lived plasma cells through CD38, CD138, and B-cell maturation antigen (BCMA). Tier 3 is a conceptual and largely investigational tier aimed at reducing relapse risk by modulating cytokine axes, including BAFF- and APRIL-dependent survival signaling, and by reinforcing immune checkpoint programs across innate and adaptive effectors.

Disruption of keratinocyte adhesion produces acantholysis, which underlies the flaccid blisters, erosions, and positive Nikolsky sign that typify the pemphigus family (1). At the junctional level, desmoglein specific IgG binds desmosomes, induces desmoglein clustering and internalization, and activates mitogen-activated protein kinase (MAPK)-dependent cytoskeletal remodeling, leading to depletion of surface cadherins and loss of cell–cell adhesion (**Figure 1A**). Pemphigus vulgaris (PV) and pemphigus foliaceus (PF) account for most cases (2). In PV, suprabasal acantholysis favors mucosal erosions with or without accompanying skin involvement. In PF, more superficial cleavage produces fragile, crusted erosions without mucosal disease (3). Paraneoplastic pemphigus/paraneoplastic autoimmune multiorgan syndrome (PNP/PAMS) marks a more complex state, with broad autoantibody profiles against desmogleins and plakin family proteins, stomatitis, and systemic manifestations such as bronchiolitis obliterans (4, 5). IgA pemphigus (IGAP) illustrates a related but distinct pattern with IgA autoantibodies and incompletely understood subcorneal pustular dermatosis and intraepidermal neutrophilic subtypes (6). Although clinical, serologic, and immune-profiling studies support a shared template of desmosomal targeting, these entities differ in effector pathways, tissue distribution, and treatment responsiveness.

## The evolution of pemphigus therapeutics and enduring obstacles

Historically, treatment relied on systemic corticosteroids with or without traditional steroid-sparing immunosuppressants (7). These regimens suppress broad immune activity and can induce remission, but chronic exposure carries substantial systemic toxicity (8). Mechanistic understanding of pemphigus has shifted therapy towards more selective approaches. For instance, anti-cluster of differentiation (CD)-20 monoclonal antibodies deplete B-cell lineages that give rise to autoantibody-producing cells and achieve remission in PV and PF (9). Randomized trial follow-up and clinical outcome data support rituximab, in combination with short-course systemic glucocorticoids, as a first-line approach for moderate to severe pemphigus vulgaris (9). Despite these advances, real-world cohort studies indicate that repeated rituximab cycles do not reliably induce durable drug-free remission and relapse rates remain significant, ranging from 11% to 44% depending on dosing, follow-up duration, and patient factors (9–12). Safety concerns further constrain indefinite B-cell depletion, with trials such as PEMPHIX showing higher rates of adverse events with repeated cycles (13, 14). As a result, reliance on repeated depletion or chronic nonspecific immunosuppression is unlikely to be sustainable and does not consistently convert remission into immune tolerance. These limitations motivate strategies that target the structure of the autoimmune network rather than only its most accessible CD20 node.

## A triadic immunity framework for the pemphigus family

A type 1, type 2, and type 3 immunity framework provides a useful conceptual lens for organizing dominant immune patterns within the pemphigus family (**Figure 1B**). In the general effector-immunity literature, type 1 responses are driven by T helper 1 cells (Th1) and group 1 innate lymphoid cells (ILC1s), both of which produce copious levels of interleukin (IL)-2, IL-12, interferon (IFN)-γ, and tumor necrosis factor (TNF)-α. They operate within an IL-12 and TNF-α rich milieu and favor IgG1/IgG3 class switching and strong complement component 3 (C3) fixation (15). Type 2 responses are driven by Th2 cells and ILC2s, which promote IgG4 class switching and eosinophil recruitment through the production of IL-4, IL-5, IL-13, and IL-31 (16, 17). These drivers are responsive to epithelial alarmins including thymic stromal lymphopoietin (TSLP), IL-25, and IL-33 (18, 19). Type 3 responses center on Th17 cells and ILC3s, with IL-17, IL-22, and IL-23 supporting a neutrophil-rich response and promoting class switching to IgG2, IgG3, and IgA; as well as weak C3 fixation (15). For each of the three branches, the same program type can be carried by Th cells alone, the ILCs alone, or both acting in concert. Moreover, these axes are mutually antagonistic, with each type suppressing one another and competing for overlapping cytokines and transcriptional spaces, although mixed states may also emerge (15).

PV and PF often occupy this landscape as type 2-skewed immune states, with programs that favor high affinity IgG4 class switching and a relatively complement sparing profile (20, 21). IgE autoantibodies have been reported in some PV and PF patients, but their pathogenic role remains to be elucidated (22, 23). By contrast, type 1-skewing is particularly relevant in PNP/PAMS, where lichenoid and interface damage and bronchiolitis obliterans point to type 1 cytotoxic effector circuits (24, 25). Type 3-skewed responses are most prominent in IGAP and other neutrophil-rich variants (20, 26, 27). The stronger type 1 and type 3 networks in PNP/PAMS and IGAP may contribute to differentiation pathways that are more resistant to CD20-directed depletion, although additional direct comparative studies are needed (8, 28). Overall, these positional biases (**Figure 1B**) across the different variants shape how B-cell tolerance is breached, how tissue inflammation is sustained, and where therapeutic interventions can be targeted with greatest precision.

Importantly, the triadic immunity framework is intended to capture dominant immune tendencies rather than impose rigid categorical boundaries. Within this structure, the relative predominance of type 1, type 2, and type 3 immune networks may be reconfigured over time by environmental or therapeutic cues, differentially organized across lesional microenvironments within the same patient, or variably expressed among individuals sharing the same clinical diagnosis (29–34). Consequently, even within a single pemphigus variant, some patients may exhibit overlapping or shifting immune programs rather than a uniform immunologic profile (30, 35–40). These immunologic differences may also be mirrored clinically by variation in mucosal and cutaneous involvement, anatomic distribution, disease severity, and treatment responsiveness (8, 41–43). Accordingly, the triadic immunity framework is best leveraged as an interpretive scaffold for discerning the prevailing immune architecture amid temporal, spatial, and interindividual heterogeneity, particularly in pemphigus vulgaris.

## Breakdown of tolerance in pemphigus

Designing network-based tolerance strategies requires a clear understanding of how B-cell tolerance is normally established and how checkpoint regulation breaks down at different stages in pemphigus.

The breadth of the antibody repertoire in health comes at the cost of maintaining a background of low affinity self-reactivity. During homeostasis, central tolerance in the bone marrow eliminates strongly autoreactive B-cell clones through apoptosis or receptor editing, while weaker self-reactive clones are permitted to exit into the periphery (44). At peripheral checkpoints, transitional B-cells in the spleen and secondary tolerance mechanisms within germinal centers (GCs) further eliminate autoreactive clones that acquire heightened self-affinity through somatic hypermutation (44). Any autoreactive cells that persist in the periphery typically remain quiescent in the absence of inflammatory activation signals (8, 44–46).

Among pemphigus variants, insights from B-cell tolerance biology, genetic susceptibility studies, and environmental risk factors collectively suggest that regulatory mechanisms may be destabilized by genetic and environmental pressures operating within type 1-, type 2-, or type 3-biased immune environments. Human genetic association studies support that susceptibility alleles in human leukocyte antigen (HLA) class II may enhance antigen presentation of desmoglein peptides and increase the likelihood that desmoglein-specific T and B-cells are recruited into productive immune synapses (8, 33). In PV, environmental stresses can induce epithelial alarmins that may drive type 2 polarized responses, while raised levels of B-cell activating factor (BAFF) and a proliferation inducing ligand (APRIL) relax B-cell survival thresholds by signaling through BAFF receptor, transmembrane activator and CAML interactor (TACI), and B-cell maturation antigen (BCMA) along a shared survival axis (47–49). Under these conditions, desmoglein-specific B-cells that would otherwise remain quiescent receive cytokine support to enter GC, undergo affinity maturation, and differentiate into memory B-cells and plasma cells.

## The adaptive network architecture of pemphigus, B-cell niches, and persistence of autoreactivity

### Networked autoreactivity and loss of immune checkpoints

Once tolerance has been breached, human lesional T/B-cell studies and integrative single-cell analyses support a model in which pemphigus autoreactivity is sustained by a distributed network linking B-cells, keratinocytes, helper T-cells, and tissue resident adaptive and innate effectors across lymphoid organs, skin, and bone marrow (**Figure 1C**) (29, 36). Based on canonical B-cell immunology and pemphigus B-cell studies, autoreactive B-cells not only secrete pathogenic antibodies but also function as antigen presenting cells. Through their B-cell receptors they internalize desmoglein, process it, and present peptides to desmoglein-specific CD4 T-cells. In turn, helper T and T follicular helper cells, through contact-dependent and contact-independent interactions, license and sustain GCs, class switching, and affinity maturation (44, 47).

Human single-cell transcriptomic, immune-gene expression, and immune-checkpoint inhibitor–associated pemphigus studies suggest that regulatory brakes that normally limit these interactions, including regulatory T-cells (Tregs) and coinhibitory pathways, such as cytotoxic T-lymphocyte-associated protein 4 (CTLA-4) and programmed cell death protein 1 (PD-1), may be weakened (32, 50, 51). Classical Tregs are numerically or functionally impaired in many patients, and desmoglein-specific Treg populations detectable in healthy individuals are often diminished (32, 52–55). This loss of regulation permits T helper programs to expand and consolidate desmoglein focused memory lineages (52). Immune checkpoint inhibitor associated pemphigus eruption underscores this vulnerability (50, 56). Beyond the widely recognized PD-1 and CTLA-4 axes, several pathways may contribute to a broader checkpoint landscape in pemphigus. By analogy to soluble PD-1 changes reported in PV, soluble CD200 levels are elevated in the serum of patients with pemphigus vulgaris and reportedly correlate with disease activity (57–59). This pattern may signify a compensatory counter-regulatory response, although the precise contribution of the CD200R:CD200 axis to pemphigus pathogenesis remains unresolved. Human genetic association and gene-expression studies in pemphigus foliaceus further implicate the leukocyte receptor complex, including LAIR/LILR-related loci, raising the possibility that inherited variation within this immunoregulatory axis may shape disease susceptibility (60, 61).

Collectively, these mapped and candidate pathways delineate a layered inhibitory network, with a core of receptors supported by direct evidence in pemphigus and a second tier of mechanistically plausible but still unmapped nodes. As this network is further investigated, it should yield a rational hierarchy of checkpoints to engage, spare, or combine when designing network-based tolerance strategies.

### Chronic germinal center activity in lymphoid organs

Once desmoglein enriched GC-like reactions are established in secondary lymphoid tissues, they can persist and sometimes organize into ectopic lymphoid structures (62). These structures are analogous to those described in systemic lupus erythematosus and rheumatoid arthritis, allowing autoreactive outputs to be renewed rather than self-limiting (63). Similar B-cell-enriched inflammatory niches are increasingly recognized in other chronic inflammatory skin diseases, including hidradenitis suppurativa (64). Tolerance within these structures fails when GC-like regulatory circuits are weakened. Human pemphigus biomarker studies show BAFF-axis perturbation after therapy, and broader B-cell survival literature supports the concept that BAFF and APRIL from stromal and myeloid sources can lower the threshold for autoreactive B-cell survival (65, 66). In parallel, human lesional studies of ectopic lymphoid-like structures support the possibility that desmosomal antigens from injured skin may be chronically displayed by follicular dendritic cells (62). Over time, intramolecular and intermolecular epitope spreading within this environment broadens desmoglein focused reactivity and stabilizes autoreactive B-cell lineages that feed the chronic pemphigus network (8, 67).

### Skin niches and *in situ* B-cell activation

Within skin, lesional and perilesional sites harbor CD4 tissue-resident memory T cells (TRMs) that, together with B-cells and plasmablasts, can support *in situ* activation (31, 36). Keratinocyte damage releases desmosomal components that are captured by dendritic cells and macrophages and presented to TRMs in skin and draining lymph nodes (68). These T-cells provide costimulatory signals and cytokines to infiltrating B-cells, promoting local class switching and plasmablast differentiation in niches at the epidermal dermal interface (29, 35, 36). In PV and PF, this circuit may reinforce the type 2, IgG4-biased programming, whereas in PNP/PAMS and IgA pemphigus it may align with their type 1 and type 3 dominated profiles. The balance within this circuit likely differs between mucosal-dominant and mucocutaneous PV; as well as subcorneal pustular dermatosis and intraepidermal neutrophilic IgA pemphigus subtypes. Persistent, low-grade epithelial injury maintains antigen availability, alarmins, and danger signals, which reinforces a feed-forward loop of local autoantibody production and tissue damage. The longevity of TRMs in these niches offers a cellular basis for focal relapse even when circulating leukocyte pools and serum biomarkers appear normalized (69).

### Long-lived plasma cell niches in bone marrow and tissue

Long-lived plasma cells (LLPC) may add a further layer of persistence in pemphigus, although direct mapping of defined LLPC niches in pemphigus remains limited. A subset of desmoglein-specific plasmablasts can differentiate into LLPCs that home to bone marrow and, in some cases, inflamed tissues (**Figure 1C**) (48, 70). These cells typically lack CD20, downregulate CD19, and upregulate CD38, CD138, and receptors for BAFF and APRIL including BCMA and TACI (71, 72). Human bone marrow plasma-cell studies and B-cell survival literature indicate that LLPCs reside in specialized stromal niches where survival signals are concentrated, including IL-6, BAFF, APRIL, and chemokines such as CXCL-12 (72). Within these niches, LLPCs can act as reservoirs of refractory autoantibody production and represent the downstream end of the survival axes (73). Because LLPCs are largely CD20-negative and CD19-low, they are insensitive to CD20 directed or CD19 directed B-cell depletion, contributing to persistent desmoglein-specific titers in some patients (70). Provided protective humoral immunity can be preserved, experience from related autoimmune conditions suggests that deeper B-cell targeting, together with rewiring of the B-cell survival signaling, may be required in select patients with anti-CD20 resistant autoimmunity (73, 74).

### Recirculating memory B-cells and relapse risk

Beyond LLPCs, recirculating memory B-cells add another dynamic component to relapse risk. After priming, desmoglein-specific memory B-cells circulate between blood and lymphoid organs and can rapidly re-expand upon re-exposure to antigen (75–77). Data from related diseases suggest that early repopulation of autoreactive memory B-cells in supporting niches tracks with clinical relapse (62, 75–77). Therefore, durable tolerance in pemphigus will likely require coordinated targeting of desmoglein-specific memory B-cells, LLPCs, and their respective niches as complements to current CD19/CD20-targeted depleting regimens.

## Innate immune circuits in pemphigus

Innate immunity pathways provide the inflammatory context in which autoreactive B- and T-cells are primed, expanded, and sustained (78, 79). In the skin, keratinocytes function as immune sentinels: they express pattern recognition receptors, sense environmental and tissue stress, and rapidly release alarmins that activate tissue resident immune cells, organizing local innate circuits that bias type 1, type 2, or type 3 downstream responses (80, 81). These innate immunity nodes therefore provide therapeutic entry points that can reduce tissue injury and lower the antigenic drive that sustains autoimmunity.

### Dendritic cells

Human lesional single-cell studies and HLA genetic association data support positioning skin-resident DCs, including epidermal Langerhans cells and dermal conventional DCs, at the interface of HLA-linked genetic risk and local epithelial injury (36, 82). Langerhans cells form a dense network within the suprabasal epidermis, whereas dermal DC subsets cluster around superficial vascular plexuses and adnexal structures in the papillary and upper reticular dermis. Human HLA association and epitope-focused studies support that, in individuals carrying pemphigus associated HLA class II alleles, such as HLA-DRB104:02, HLA-DRB114, HLA-DQB103:02, and HLA-DQB105:03, the peptide binding groove preferentially accommodates defined desmoglein 3 and desmoglein 1 epitopes (83). Human desmoglein epitope studies and mechanistic DC-trafficking models support the possibility that, under these conditions, skin DCs are more likely to present desmoglein peptides in draining lymph nodes and to sustain desmoglein-specific adaptive responses (40, 84). Strategies that promote tolerogenic DC phenotypes or deliver desmoglein antigens in non-inflammatory contexts, including nanoparticle-based tolerizing vaccines or engineered DCs as developed in type 1 diabetes and inflammatory bowel disease, may redirect priming from effector responses toward induction of desmoglein-specific Tregs (85, 86).

### Macrophages

Dermal macrophages inhabit perivascular and periadnexal niches and continuously clear debris and immune complexes (87–89).

Pemphigus lesional transcriptomic data, together with broader macrophage and Fc-receptor biology, support that macrophages in pemphigus lesions may bind desmoglein IgG-containing immune complexes via Fc receptors, engulf apoptotic keratinocytes, and release inflammatory mediators such as IL-1, IL-6, and TNF-α (89–93). This amplifies local inflammation and recruits additional effector cells. Within inflamed skin, macrophages also produce BAFF and APRIL, positioning them as a stromal–myeloid node that couples ongoing tissue damage to a microenvironment favoring persistence of the autoreactive B-cell repertoire and supporting plasmablast and plasma cell survival (62, 89).

### Innate lymphoid cells

Innate lymphoid cells (ILCs) coordinate type 1, type 2, and type 3 immunity. In human skin, ILC2s have been described clustering in the superficial dermis with extensions toward the lower epidermis, positioning them to sense barrier disruption and mechanical stress (94, 95). ILC1s and ILC3s are enriched within inflamed dermis and epidermis in autoimmune skin disease. In PV, emerging human cohort studies report increased circulating ILC1 and ILC3 populations (96, 97). ILC1 frequency has been reported to correlate with anti-desmoglein 3 titers and clinical severity, and ILC3-associated type 3 cytokine signaling has been reported in lesional skin of some patients (89, 97). These observations are consistent with a model in which ILC subsets translate epithelial stress into programs that license local cutaneous inflammation and may help sustain autoreactive B-cell and T-cell responses. Additionally, these findings emphasize that non–type 2 innate programs can coexist with type-2-associated IgG4-skewed autoantibody biology across PV patients, lesions, or disease stages.

Several established checkpoints discussed above, including PD-1, CD200R, and LAIR-1 are expressed on ILCs in barrier tissues (16–19, 98–102). By analogy to other autoimmune settings, this raises the possibility that checkpoint erosion or compensatory upregulation in pemphigus also involves ILC programs and contributes to dysregulated effector responses. Clinical use of dupilumab in refractory pemphigus remains empirical and limited to case reports and small case series, but reported steroid-sparing responses suggest that type 2 pathways, potentially including ILC2 dependent circuits, may modulate the pemphigus network (103–105). Taken together, these observations position ILCs as strategically placed and plausible nodes within the pemphigus network that warrant further investigation as candidate targets for future network-based tolerance strategies.

### Natural killer cells

Natural killer (NK) cells patrol the dermis and perivascular spaces and may provide an Fc receptor-dependent arm within the pemphigus network (103, 106). Leveraging CD16, they recognize IgG-coated targets and may contribute to antibody-dependent effector injury, particularly in the more type 1-skewed context of PNP/PAMS (5, 107, 108). NK-cell production of IFN-γ and TNF-α may reinforce this milieu, but their contribution in pemphigus remains incompletely defined.

## Shifting the therapeutic paradigm toward network-based tolerance

A type 1, type 2, and type 3 immunity framework of the pemphigus family supports a therapeutic model that moves beyond broad immunosuppression toward deliberate immune engineering. Immediate suppression of disease activity remains essential to prevent morbidity and mortality, but the broader objective is to rewire the immune system toward a new equilibrium in which desmoglein-specific responses remain durably quiescent after suppression is withdrawn (9, 109).

To this end, treatment can be viewed as a three-tiered network-based strategy (**Table 1**). The first tier, which largely reflects current clinical practice, interrupts effector mechanisms by depleting autoreactive B-cells and lowering pathogenic antibodies, with emerging CD19-directed CAR-T approaches, including CABA-201, representing a more intensive extension of this lineage-depleting strategy (110). The second tier remains investigational and focuses on targeting plasmablasts and LLPCs in select patients with anti-CD20-resistant autoimmunity. The third conceptual tier aspires to prevent relapse by strengthening tolerogenic networks, directing circulating memory B-cell programming, and interrupting pathogenic TRM and innate circuits in skin and mucosa (**Figure 1D**).

**Table 1:** Network-based immunotherapeutic tiers in pemphigus.

Tier	Therapeutic objective(s)	Target node(s)	Representative strategies	Main rationale	Main limitation/risk
Tier 1: Effector B-cell depletion	Disease control by rapidly suppressing active disease and reducing ongoing pathogenic anti-desmoglein antibody generation.	Upstream B-cell pool: naive B cells, germinal center B cells, memory B cells, and some plasmablasts.	Anti-CD20 depleting antibodies or CAR-T platforms. Anti-CD19 depleting antibodies or CAR-T platforms. Anti-desmoglein-specific B-cell receptor (dBCR), anti-CD20 (or CD19) bispecific antibodies or CAR-T platforms. DSG3-CAART platforms.	Interrupts effector mechanisms by removing autoreactive B-cell precursors and lowering new autoantibody output.	Does not reliably eliminate plasmablast and LLPCs, which may be CD19 or CD20 low/negative.
Tier 2: Deep plasmablast and long-lived plasma cells (LLPC) targeting	Disease control in relapsing, refractory, tier 1 resistant cases	Plasmablasts and LLPCs supported by lymph node and bone marrow stromal niches.	Anti-CD38 depleting antibodies or CAR-T platforms. Anti-CD138 depleting antibodies or CAR-T platforms. Anti-BCMA depleting antibodies or CAR-T platforms. Anti-CD3, anti-BCMA (or CD38, or CD138) bispecific T-cell engagers. Anti-dBCR, anti-BCMA (or CD38, or CD138) and bispecific antibodies or CAR-T platforms. DSG3-CAART platforms.	Extends depletion beyond CD20-positive B cells to the plasma-cell compartments that can sustain autoantibody production and cause relapse after tier 1-based B-cell depletion strategies.	Progressively deeper B-cell/plasma-cell depletion can compromise protective humoral immunity, including hypogammaglobulinemia and loss of vaccine-derived immunity. Some plasmablast and LLPCs may downregulate surface BCR.
Tier 3: Network reprogramming and relapse prevention	Establish durable immune tolerance after tier 1- or 2-based treatment	Cytokine, stromal, innate, and checkpoint circuits and survival pathways across skin, mucosa, lymph node, and bone marrow	Anti-BAFF blocking antibodies. Anti-APRIL blocking antibodies. Dual BAFF/APRIL blockades (eg, TACI-Fc decoy receptor Telitacicept). Cytokine blockades (eg, anti-IL-6, anti-IL-4Rα). Immune Checkpoint Restoration (eg, PD-1 agonists, CTLA-4 agonists).	Shifts treatment from depletion alone toward a new immune equilibrium in which desmoglein-specific responses remain quiescent after suppression is withdrawn.	Mostly conceptual or investigational in pemphigus; requires biomarkers to determine which survival, cytokine, checkpoint, innate, or stromal nodes drive relapse in each patient.

The survival networks that underpin tiers two and three already anchor much of the next generation of therapeutics across various autoimmune conditions (111–113). Telitacicept, a TACI Fc fusion that neutralizes both BAFF and APRIL, was recently approved in China for systemic lupus erythematosus, illustrating the feasibility of targeting upstream survival signals (111). Further downstream, anti-BCMA depleting antibodies and BCMA/CD3 bispecific T-cell engagers may enable deeper plasma-cell targeting (113–115). However, progressively deeper B-cell depletion has been accompanied by loss of vaccine-derived immunity and hypogammaglobulinemia in other autoimmune contexts (115). Our current understanding of pemphigus biology may offer a way out of this constraint. Unlike lupus and Sjögren disease, the dominant pathogenic antigens in pemphigus, desmoglein 1 and 3, are well defined and map onto discrete desmoglein-specific B-cell receptors (dBCR). This creates an opportunity for antigen-selective immune engineering, exemplified by RESET-PV (NCT04422912), which evaluates autologous DSG3-CAAR-T therapy in active pemphigus vulgaris (116). Related strategies, such as bispecific anti-CD20/anti-desmoglein-specific B-cell receptor depleting antibodies, could in principle couple lineage markers to autoreactive B-cell receptor specificity, thereby eliminating pathogenic B-cell-lineage populations while sparing protective humoral immunity. Across all pemphigus variants, truly antigen selective deep B-cell targeting, together with remodeling of stromal and innate niches will be central to allow a non-autoreactive B-cell repertoire to reemerge within a more balanced immune landscape.

## Concluding remarks

The biology of pemphigus illustrates how organ-specific autoimmunity is sustained not by autoreactive B cells in isolation, but by an integrated immune network spanning adaptive lymphocytes, innate effectors, and tissue niches across the skin, lymphoid organs, and bone marrow. Viewed through a network-based lens, the therapeutic paradigm shifts beyond empiric immunosuppression toward precise reprogramming of the autoimmune response through modulation of innate circuits, rewiring of survival pathways, and restoration of antigen-specific tolerance, ultimately enabling long-term, drug-independent remission.
